# EM-driven size reduction and multi-criterial optimization of broadband circularly-polarized antennas using pareto front traversing and design extrapolation

**DOI:** 10.1038/s41598-022-13958-9

**Published:** 2022-06-14

**Authors:** Ubaid Ullah, Muath Al-Hasan, Slawomir Koziel, Ismail Ben Mabrouk

**Affiliations:** 1 Al Ain University, P.O. Box (112612), Abu Dhabi, United Arab Emirates; 2 Engineering Optimization and Modeling Center, Reykjavik University, 102 Reykjavik, Iceland; 3 Faculty of Electronics, Telecommunications and Informatics, Gdansk University of Technology, 80-233 Gdansk, Poland; 4 Department of Engineering, Durham University, Durham, DH1 3LE UK

**Keywords:** Engineering, Electrical and electronic engineering

## Abstract

Maintaining small size has become an important consideration in the design of contemporary antenna structures. In the case of broadband circularly polarized (CP) antennas, miniaturization is a challenging process due to the necessity of simultaneous handling of electrical and field properties (reflection, axial ratio, gain), as well as ensuring sufficient frequency range of operation, especially at the lower edge of the antenna bandwidth. An additional difficulty is that—for the sake of reliability—the design process has to be based on full-wave electromagnetic simulation tools. This is a computationally expensive endeavor because rendering the minimum-size design under the assumed constraints concerning the operating frequencies requires rigorous numerical optimization, which entails massive evaluations of the structure at hand. This paper describes an algorithmic framework for efficient identification of broadband CP antenna designs that realize the best possible trade-offs (Pareto set) between the antenna size and its operating bandwidth. The designs are generated sequentially by solving local optimization tasks targeting explicit reduction of the antenna footprint with implicit constraints imposed on the reflection and axial ratio characteristics. The data accumulated during the previous iterations is employed to yield good initial points for further stages by means of inverse surrogates and extrapolation. Low cost of the process is ensured by sparse sensitivity updates within the trust-region gradient-based algorithm being the main optimization engine. The proposed methodology is demonstrated using three examples of wide-slot CP structures with the trade-off designs representing broad ranges of achievable antenna sizes and operating bandwidth. The framework can be used to assess the antenna suitability for particular application areas as well to conclusively compare alternative CP geometries from the point of view of achievable miniaturization rate and capability of fulfilling given performance requirements.

## Introduction

Rapidly growing wireless communication technology fostered serious attention towards broadband circularly polarized (CP) antennas. Circular polarization has the advantage of minimizing the multipath effects, the Faraday’s effect, polarization mismatch, and the absorption losses. In addition to this, CP antennas enable stability of the communication links, as well as the flexibility in the positioning angle between the transmitter and the receiver^1,2^ Owing to these features, the implementation of circular polarization in the modern broadband wireless systems has been extensively researched^3,4^.

In general, simultaneous excitation of two orthogonal field components with equal magnitudes is required to attain circular polarization. By using two feeding sources, CP can be easily achieved but this technique is not useful for implementing compact antennas because of the added complexity. Alternatively, a single feeding source may be utilized in conjunction with the topologically modified structures, power dividers, or sequentially rotated feeding structures. For instance, various realizations of CP antennas have been proposed, in which either the geometry of the slot, the ground plane, and/or the feeding line have been altered^5,6^. The geometrically modified structures are largely based on planar geometries, which include microstrip patches and printed slots. The metallic patch type antennas are generally narrowband and can serve one purpose at a time, which limits their applications in broadband communication systems^7^. On the other hand, the printed slot-type antennas are capable of operating over a wide frequency range, therefore have attracted attention of the researchers and antenna engineers^8,9^.

For the wide-slot antennas, in particular, the compromise between the gain and the bandwidth is achieved by removing parts of metal from the radiator^10^. Geometrically, modified planar wide slots, patches, and strip-line antenna structures can meet the stringent requirements concerning broadband performance while maintaining acceptable electrical and field characteristics^11^. Nevertheless, miniaturization of the geometrically modified CP antenna is a serious challenge due to non-linear relations between the distributed field components on the structure and the radiated fields. The operating frequency and polarization of the radiated fields are dependent on multiple parameters. In particular, miniaturization of such electromagnetically and topologically complex structures requires seeking for trade-offs between the antenna performance in terms of electrical characteristics and size. Furthermore, in the case of broadband CP antenna designs, several characteristics have to be taken into consideration, including impedance matching, axial ratio, but also operating bandwidth.

Regardless of a particular set of objectives considered in the design process, identification of the compromise solutions requires multi-objective optimization (MO)^12^. MO aims at generating a family of designs representing the best possible trade-offs between the objectives, also referred to as a Pareto set^13^. From the perspective of numerical optimization algorithms, MO is significantly more challenging than single-objective optimization already at the stage of comparing the designs, which is typically based on Pareto dominance relation^14^ For convenience, multi-objective problems are often transformed into single-objective ones using, e.g., objective aggregation (weighted-sum method^15^]), or prioritization (selecting the primary objective and handling the remaining ones using appropriately defined constraints^16^_._ This allows for the employment of a large variety of available algorithms, both local^17^,and global^18–20^. On the other hand, genuine MO procedures permit the rendition of the entire Pareto set at a single algorithm execution^21^. The most popular solution approaches are nature-inspired population-based procedures^22–25^. However, their computational cost is high, which hinders direct handling of EM simulation models, otherwise mandatory to ensure reliable evaluation of antenna characteristics. Possible workaround includes surrogate-assisted methods, where the metaheuristic procedures operate on fast metamodels (e.g., kriging^26^, Gaussian process regression^27^, or neural networks^28^). The surrogates can be constructed within the entire parameter space (only for low-dimensional problems^29^), or iteratively refined using sequential sampling techniques^30,31^. In either case, only relatively simple cases characterized by up to a few (four to six) parameters are normally reported^32,33^. Performance-driven modeling methods are capable of overcoming the dimensionality issue to a much larger extent^34,35^, just as some recent deterministic approaches (e.g., sequential domain patching^36^, or generalized bisection algorithm^37^).

This paper proposes a simple yet efficient algorithmic approach to generating trade-off solutions for broadband CP antennas, in particular, the family of Pareto-optimum points that represent minimum-size designs for a sequence of target operating bandwidths. Availability of such solutions equips the designer with invaluable know-how concerning the capability of a given antenna structure, its suitability for size-limited applications, but also allows for meaningful comparison with alternative antenna geometries. According to the presented methodology, the trade-off designs are obtained sequentially by solving local optimization tasks formulated as explicit footprint reduction with the constraints imposed on the reflection and axial ratio characteristics. The constraints are handled using penalty functions, which permit the employment of single-objective optimization procedures. At the same time, iterative adjustment of the target bandwidth allows for traversing the Pareto front. To speed up the optimization process, the data accumulated during the prior iterations is used to produce good-quality initial points for further stages. This is realized using inverse modeling methods^38^ and extrapolation. Furthermore, the low cost of the parameter tuning process is ensured by utilization of trust-region gradient search with sparse sensitivity updates^39,40^. The proposed technique is demonstrated using three examples of wide-slot CP structures. In each case, the family of compromise designs is found for broad ranges of achievable antenna sizes and operating bandwidths.

The novelty and the technical contributions of the paper can be summarized as follows: (1) the development of a novel deterministic approach for generating a family of minimum-size designs of broadband CP antennas parameterized by the target operating bandwidth, (2) incorporation of the inverse modeling methods and other algorithmic approaches to generate initial designs and expedited parameter tuning, (3) demonstration of a practical design utility of the proposed methodology using several wide-slot CP antennas, as well as a possibility of significant reduction of antenna footprint while maintaining tight control over the impedance and axial ratio bandwidth. A possible application of our procedure is the assessment of the antenna suitability for particular application areas as well as a conclusive comparison of alternative CP antenna geometries from the point of view of achievable miniaturization rate and capability of fulfilling given performance requirements.

## Broadband circularly polarized antennas: design challenges

To meet the requirements of broadband operation and ease of integration, a large number of CP antennas with planar structures have been proposed in the literature^2,41–45^. On the one hand, broadband operation with circular polarization can be attained using the well-known wide-slot type of antennas. Nevertheless, the design process of such structures is quite challenging due to the geometrical complexity associated with this class of antennas. In general, the shape of the slot can be arbitrary, which makes any predictions concerning the operating frequency of the antenna difficult due to the lack of theoretical models or systematic design procedures. Traditionally, these antennas have been developed based on heuristic approaches combined with the tedious multi-stage analysis of the simulated surface field distribution. The latter involves computationally demanding full-wave simulations, which increase the CPU cost of the design process.

**Figure 1:**
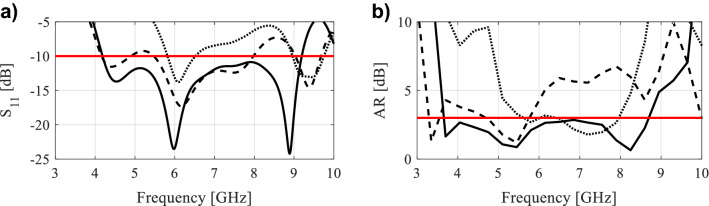
Rudimentary scaling of an exemplary wide-slot CP antenna: the design optimized for 4.2 GHz (solid line), antenna responses upon scaling to 3.7 GHz (dashed line), antenna responses upon scaling to 4.5 GHz (dotted line): (**a**) |S11|, (**b**) AR.

Following the initial sizing of the antenna, the geometry is further optimized to operate within the frequency band of the target applications. This is usually done through supervised parameter sweeping. For the majority of the antennas designed using such methods^46^, the impedance bandwidth is not aligned with axial ratio (AR) bandwidth. Consequently, the structure exhibits circular polarization in some parts of the antenna operating band, and linear polarization in the remaining part. Furthermore, due to the arbitrarily shaped slot, the number of adjustable parameters is normally large, whereas the antenna characteristics (especially axial ratio) are extremely sensitive to these variables. Even relatively small changes in antenna dimensions may significantly affect both the impedance matching and AR. To illustrate this, a simple wide-slot antenna is designed and optimized for the lower operating frequency of roughly 4.2 GHz, and taken as a reference design. Figure 1 shows the antenna impedance matching and AR when modifying the antenna parameters.

This indicates that the redesign of such structures requires meticulous and simultaneous adjustment of their geometry parameters, which can only be done through numerical optimization. The problem is grossly aggravated when antenna miniaturization is of interest, in which case electrical and field characteristics have to be adjusted to conform to a particular operating band while explicitly reducing the antenna size. This requires the development of a rigorous optimization framework, being the very subject of this work.

## Algorithmic framework for antenna miniaturization: generating size-bandwidth trade-off designs

This section introduces the proposed algorithmic approach to fast identification of the best possible CP antenna design trade-offs. Theseare understood as the family of designs that represent the structures featuring the minimum footprint areas for given target values of the operating bandwidths. The designs are generated sequentially using a combination of knowledge-based predictions from already existing designs, and local parameter tuning arranged using trust-region gradient search with sparse sensitivity updates. We start by formulating the design task, followed by the description of the main components of the procedure: initial design rendition using inverse surrogates, and gradient-based parameter tuning. The entire framework is summarized in. The methodology is illustrated below using several examples of broadband wide-slot CP antennas.

## Size reduction of CP antennas: problem formulation

A vector of designable (geometry) parameters of the antenna will be denoted as ***x*** = [*x*_1_ … *x*_*n*_]^*T*^. The EM-simulated reflection and axial ratio responses are *S*_11_(***x***,*f*) and *AR*(***x***,*f*), respectively, where *f* stands for the frequency. In this work, the objective is to find the minimum-size designs corresponding to the pre-defined operating bandwidths of the circularly-polarized antennas. The operating bandwidth *B* is determined by the lower and upper frequencies *f*_*L*_ and *f*_*H*_, i.e., *B* = [*f*_*L*_*f*_*H*_], and the antenna is supposed to satisfy the following two conditions

1 $$|S_{11} ({\mathbf{x}},f)| \le - 10\;{\text{dB}}\;\;{\text{for}}\;\;f_{L} \le f \le f_{H}$$

and

2 $$|AR({\mathbf{x}},f)| \le 3\;{\text{dB}}\;\;{\text{for}}\;\;f_{L} \le f \le f_{H}$$

The antenna footprint area will be denoted as *A*(***x***). It is supposed to be reduced as much as possible while ensuring that both (1) and (2) hold. To handle this task, given *f*_*L*_ and *f*_*H*_, we define the following cost function that is to be minimized:

3 $$U({\mathbf{x}},f_{L} ,f_{H} ) = A({\mathbf{x}}) + \beta_{S} c_{S} ({\mathbf{x}},f_{L} ,f_{H} )^{2} + \beta_{AR} c_{AR} ({\mathbf{x}},f_{L} ,f_{H} )^{2}$$

where

4 $$c_{S} ({\mathbf{x}},f_{L} ,f_{H} ) = \max \left\{ {\max \left\{ {f_{L} \le f \le f_{H} :|S_{11} ({\mathbf{x}},f)| + 10} \right\},0} \right\}$$

5 $$c_{AR} ({\mathbf{x}},f_{L} ,f_{H} ) = \max \left\{ {\max \left\{ {f_{L} \le f \le f_{H} :AR({\mathbf{x}},f) - 3} \right\},0} \right\}$$

In (3), our primary goal is the reduction of the antenna size, whereas the conditions (1) and (2) are enforced implicitly using the penalty terms. The functions *c*_*S*_ and *c*_*AR*_ quantify possible violations of the reflection and axial ratio requirements and contribute to the cost function if and only if such a violation has been detected. The amount of contribution is controlled using the coefficients *β*_*S*_ and *β*_*AR*_, which also allow us to determine how much violation can be tolerated. In our numerical experiments, we set *β*_*S*_ = *β*_*AR*_ = 100. For these values, 1 dB violation of either (1) or (2) increases the value of the cost function (3) by 100, which is significant given that the typical footprint areas (measures in mm^2^) are a few hundred. However, violations at the level of 0.3 dB or so, increase the cost function by around 10, which is noticeable, yet may occur at the optimized design as a compromise between size reduction and constrain satisfaction.Thus, effectively, the penalty coefficients play a role in the normalization factors. It should also be noticed that the penalty functions are squared in the objective function (3). This is to ensure that the objective function is smooth with respect to the amount of constraint violation, which facilitates exploration of the feasible region boundary (if squares are not used, the objective function would be non-differentiable at the boundary of the feasible region).

Using the objective function (3)–(5), the design problem is formulated as

6 $${\mathbf{x}}^{*} = \arg \mathop {\min }\limits_{{\mathbf{x}}} U({\mathbf{x}},f_{L} ,f_{H} )$$

In the following sub-sections, we introduce the procedure for generating minimum-size designs [in the sense of (6)], representing trade-offs between the achievable footprint area and the operating bandwidth.

## Pareto front traversing for sequential identification of trade-off solutions: design extrapolation

Our primary objective is the reduction of the antenna footprint as well as the identification of the compromise designs that represent the best possible trade-offs between the antenna size and operating bandwidth, especially its lower end. In particular, given a sequence of the lower operating frequencies *f*_*L.k*_, *k* = 1, …, *N*, *f*_*L.*1_ < *f*_*L.*2_ < … < *f*_*L.N*_, the aim is to find the designs x^*(*k*)^, *k* = 1, …, *N*, which are optimum in the sense of (6) for *U*( ⋅,*f*_*L.k*_,*f*_*H*_), where *f*_*H*_ is the upper end of the operating band, common for the entire sequence. Recall, that solving the problem (6) with the objective function (3)–(5) ensures satisfactory levels of antenna impedance matching and axial ratio within the target operating band. As mentioned before, the availability of the trade-off solutions is important from the design perspective as it allows for assessing the antenna suitability for specific applications (especially, the space-limited ones), as well comparing alternative antenna topologies in terms of achievable miniaturization rates.

In this work, the sequence {x^*(*k*)^} is generated sequentially, starting from the design corresponding to the broadest operating band [*f*_*L*.1_*f*_*H*_]. The data obtained so far in the process is employed to generate the initial designs for further tuning. Below, a procedure for initial point rendition through design extrapolation is outlined, whereas the tuning procedure is explained.

- Design x^*(1)^: Because there is no data accumulated yet, the first design is obtained through direct optimization of the EM antenna model, here, using the local tuning algorithm given below, and starting from whatever design can be found using the standard means (e.g., parameter sweeping, etc.).
- Design x^*(2)^: The initial design ***x***^(2.0)^ is obtained by simply scaling the external dimensions of the antenna substrate by the factor *f*_*L*.2_/*f*_*L*.1_. For efficient optimization, the antenna is normally parameterized so that most of its internal structure (slots, stubs, etc.) have their dimensions relative to the substrate size. Owing to that, scaling of only the external dimensions allows for preserving the internal (size) relationships and avoiding severe degradation of the antenna characteristics (particularly, the axial ratio response) while shifting the lower operating frequency as required.
- Design x^*(3)^: The initial design ***x***^(3.0)^ is obtained using the information from both x^*(1)^ and x^*(2)^, by setting
  7 $${\mathbf{x}}^{(3.0)} = {\mathbf{x}}^{*(2)} + \frac{{f_{L.3} - f_{L.2} }}{{f_{L.2} - f_{L.1} }}\left[ {{\mathbf{x}}^{*(2)} - {\mathbf{x}}^{*(1)} } \right]$$
  In other words, linear extrapolation is employed; however, in practice, the formula (7) is only applied for the parameters controlling the external dimensions of the substrate for the same reasons as explained in the previous paragraph.
- Designs x^*(*k*)^, *k* > 3: The components of the parameter vector ***x***^(*k*.0)^ = [*x*_1_^(*k*.0)^ … *x*_*n*_^(*k*.0)^]^*T*^ are found by extrapolating the data contained in all designs x^*(*j*)^, *j* = 1, …, *k* – 1. This is done by setting up (inverse) nonlinear regression models of the form
  8 $${\mathbf{s}}_{j} (f_{L} ,{\mathbf{p}}_{j} ) = p_{j.1} + p_{j.2} \exp (p_{j.3} f_{L} ),\quad j = \, 1, \, \ldots ,n$$
  with the model coefficients found using nonlinear regression of the form
  9 $${\mathbf{p}}_{j}^{*} = \arg \mathop {\min }\limits_{{{\mathbf{p}}_{j} }} \sum\limits_{l = 1}^{k - 1} {({\mathbf{s}}_{j} (f_{L.l} ,{\mathbf{p}}_{j} ) - x_{j}^{*(l)} )^{2} } , \quad j = \, 1, \, \ldots ,n$$
  Having established the model, we get
  10 $$x_{j}^{(k.0)} = {\mathbf{s}}_{j} (f_{L} ,{\mathbf{p}}_{j}^{*} ),\quad j = \, 1, \, \ldots ,n$$

As indicated in^38,39^, exponential curves are convenient choices as an analytical form of the inverse model because they ensure sufficient flexibility while using a small number of coefficients, and represent well typical relationships between the operating frequency and antenna dimensions.

## Local tuning algorithm

The initial designs obtained as explained in earlier have to be tuned in order to reduce the antenna footprint while ensuring the satisfaction of the conditions (1) and (2) for given lower operating frequencies *f*_*L*.*k*_, which is achieved by solving the problem (6) with the objective function (3)–(5). Owing to the extrapolation techniques described above, the initial designs are normally of good quality so local tuning is sufficient. Here, it is realized using an accelerated version of the trust-region (TR) gradient-search proposed in^40^. The algorithm is briefly outlined here for the convenience of the reader.

The reference algorithm is the standard TR procedure^47^, which generates a series of approximations ***x***^(*k.i*)^, *i* = 0, 1, …, to the optimum design x^*(*k*)^ by solving sub-problems

11 $${\mathbf{x}}^{(k.i + 1)} = \arg \mathop {\min }\limits_{{{\mathbf{x}};\; - {{\varvec{\updelta}}}^{(i)} \le {\mathbf{x}} - {\mathbf{x}}^{(k.i)} \le {{\varvec{\updelta}}}^{(i)} }} U_{L} ({\mathbf{x}},f_{L.k} ,f_{H} )$$

where *U*_*L*_ is the objective function (6) computed using linear expansion models of the antenna reflection and axial ratio characteristics, defined as

12 $$S_{L}^{(k.i)} ({\mathbf{x}},f) = S_{11} ({\mathbf{x}}^{(k.i)} ,f) + {\mathbf{G}}_{S} ({\mathbf{x}}^{(k.i)} ) \cdot ({\mathbf{x}} - {\mathbf{x}}^{(k.i)} )$$

13 $$AR_{L}^{(k.i)} ({\mathbf{x}},f) = AR({\mathbf{x}}^{(k.i)} ,f) + {\mathbf{G}}_{AR} ({\mathbf{x}}^{(k.i)} ) \cdot ({\mathbf{x}} - {\mathbf{x}}^{(k.i)} )$$

In a conventional TR algorithm, the gradients ***G***_*S*_ and ***G***_*AR*_ are estimated using finite differentiation (FD); ***δ***^(*i*)^ is the trust-region size vector adjusted using the conventional TR rules^23^. The inequalities –***δ*** ^(*i*)^ ≤ ***x*** – ***x***^(*i*)^ ≤ ***δ*** ^(*i*)^ in (11) are understood component-wise.

The accelerated version of the TR procedure employed here^40^ reduces the cost of estimating the antenna sensitivities by omitting the expensive FD updates for the parameters that exhibit small gradient variability across the algorithm iterations (as compared to other parameters). The assessment is realized using an appropriate metric as described below. This mechanism results in significant (almost 50%) computational savings as compared to the reference algorithm, with minimum degradation of the design quality.

The concept of gradient monitoring is explained for the reflection response *S*_11_(***x***,*f*) but applies to *AR* as well. The gradient ***G***_*S*_ is a 1 × *n* vector with *G*_*k*_ representing the sensitivity of *S*_11_(***x***,*f*) w.r.t the *k*th parameter, *k* = 1, …, *n*. The gradients at two subsequent iterations are compared using the following metric (averaged over the frequency band of interest *F*):

14 $$d_{k}^{{\left( {i + 1} \right)}} = \mathop {mean}\limits_{f \in F} \left( {2 \cdot \frac{{\left| {G_{k}^{\left( i \right)} (f)} \right| - \left| {G_{k}^{{\left( {i - 1} \right)}} (f)} \right|}}{{\left| {G_{k}^{\left( i \right)} (f)} \right| + \left| {G_{k}^{{\left( {i - 1} \right)}} (f)} \right|}}} \right)$$

where *G*_*k*_^(*i*)^ stands for the *k*th component of ***G***_*S*_ in the *i*th iteration. The following auxiliary variables are defined: ***d***^(*i*)^ = [*d*_1_^(*i*)^ … *d*_*n*_^(*i*)^]^*T*^ – a vector of gradient difference factors (cf. (14)) in the *i*th iteration, *d*_min_^(*i*)^ = min{*k* = 1,…,*n* : *d*_*k*_^(*i*)^}, *d*_max_^(*i*)^ = max{*k* = 1,…,*n* : *d*_*k*_^(*i*)^}, and ***N***^(*i*)^ = [*N*_1_^(*i*)^ … *N*_*n*_^(*i*)^]^*T*^—a vector of the numbers of upcoming algorithm iterations FD to be omitted.

The entries *N*_*k*_^(*i*)^ are computed using user-defined the minimum and the maximum number of iterations without FD, denoted *N*_min,_ and *N*_max_. We have

15 $$N_{k}^{{(i)}} = \llbracket {N_{{\max }} + a^{{(i)}} (d_{k}^{{(i)}} - d_{{\min }}^{{(i)}} )} \rrbracket$$

where *a*^(*i*)^ = (*N*_max_– *N*_min_)/(*d*_min_^(*i*)^ – *d*_max_^(*i*)^) (here, $[[.]]$ is the nearest integer function^40^). As mentioned before, the above technique controls the frequency of using finite-differentiation updates for particular antenna parameters based on gradient variability quantified as in (14), and the maximum number of subsequent iterations without the updates (*N*_max_) is assigned to those parameters that exhibit the smallest variability, whereas the minimum number *N*_min_ is associated with the parameters for which the variability is the highest. The vector ***N***^(*i*)^ is updated in each iteration, which allows for adaptive adjustment of the FD frequency depending on the sensitivity profiles at any given stage of the optimization process.

The termination condition for the algorithm is convergence in argument ||***x***^(*i*+1)^ – ***x***^(*i*)^||< *ε* (here, *ε* = 10^–2^ is the termination threshold) or sufficient reduction of the TR size ||***δ***^(*i*)^||< *ε*, whichever occurs first. It should be noted that convergence of the process follows from the classical TR theory^47^, whereas, in practice, it is often due to the reduction of the trust-region size.

## Redesign procedure

**Figure 2:**
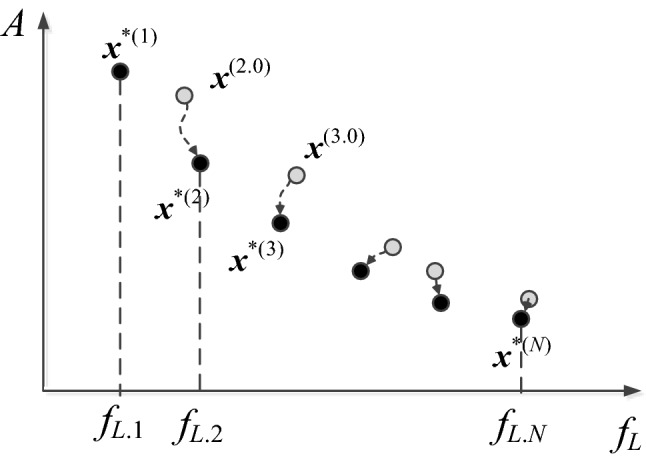
Graphical illustration of the Pareto set for CP antenna, representing the best possible trade-off solutions with respect to the lower operating frequency and antenna size. The black circles mark the final designs, whereas the gray circles are the initial points obtained using the procedure above.

The proposed optimization framework yields a family of minimum-size designs corresponding to the input (user-defined) sequence of the operating bandwidth, here, determined by the lower operating frequencies *f*_*L.k*_and a fixed upper-frequency *f*_*H*_. Figure 2 shows an exemplary Pareto set with the initial and refined designs marked using the gray and the black circles, respectively. In general, the predictions concerning the initial designs are becoming more accurate for the increased value of k because more and more already established designs are utilized in the extrapolation process.

**Figure 3:**
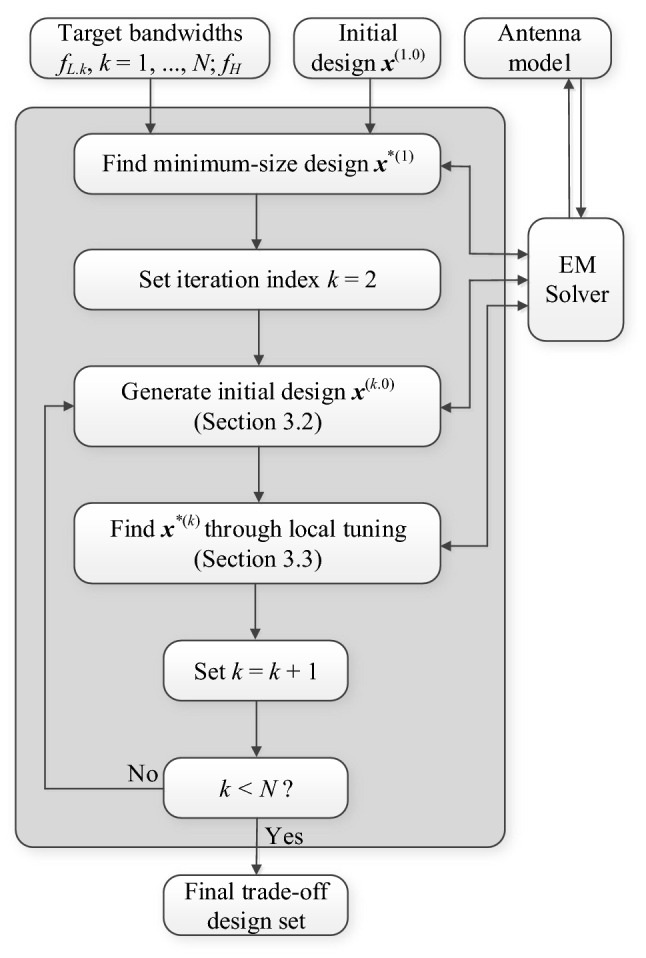
Flow diagram of the proposed procedure for generating the family of minimum-size circularly-polarized antenna design as a function of the operating band.

Figure 3 shows the flow diagram of the design process. The input parameters include the target operating bandwidths, the initial point necessary to generate the first minimum-size design, and, the computational model of the antenna at hand, here, implemented in CST Microwave Studio. The optimization algorithm is implemented in Matlab, and the communication between Matlab and CST is realized using a custom-built interface.

## Demonstration case studies

For the sake of demonstration, the methodology presented in the earlier sections has been applied to generate trade-off designs for three recent wide-slot CP antenna realizations^46,48,49^. In each case, we are searching for minimum-size designs corresponding to the pre-defined operating bands that range from about 3 GHz until 7.5 GHz. The trade-off designs obtained for the increasing values of the lower edge of the operating bandwidth (e.g., 3.0 GHz, 3.5 GHz, 4.0 GHz, etc.) essentially represent the Pareto sets from the point of view of the two figures of interest, one being footprint area reduction, and the other being the operating bandwidth for which both the impedance matching and the axial ratio satisfy the conditions |*S*_11_|≤ –10 dB and *AR* ≤ 3 dB, respectively. It should be emphasized that this section only contains numerical results. Experimental validation is not provided because all three examples considered here were previously reported in the literature^46,48,49^, as well as prototyped and measured accordingly in the respective original works.

## Example 1: Wide-slot CP antenna^46^

**Figure 4:**
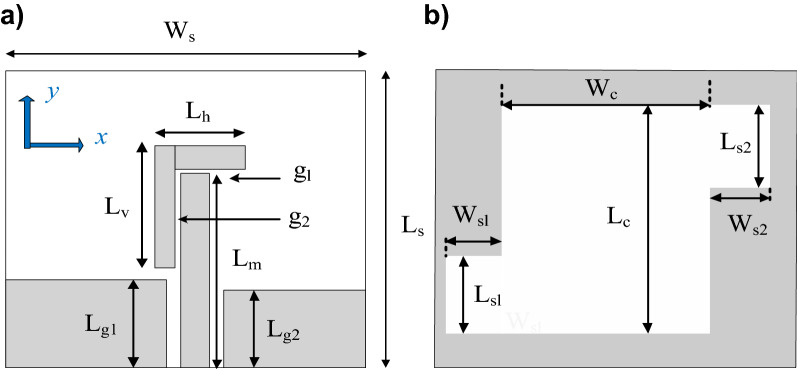
Wide-slot CP antenna^46^: (**a**) front view, (**b**) back view.

The first demonstration case study^46^, is shown in Fig. 4, and implemented on Rogers RO4003C substrate (*ε*_*r*_ = 3.38, tan*δ* = 0.0027, *h* = 0.813 mm). The reference design shown in Fig. 4 is designed on a double-sided substrate. The coplanar gap *g*, and the separation between the feedline and the parasitic strip along the horizontal and vertical plane are fixed. The microstrip feedline used for excitation of the antenna and the L-shape parasitic strip placed in the vicinity of the feedline generates the orthogonal components required for the fundamental CP mode. The AR bandwidth is further widened by the geometrically modified slot in the backside ground plane by exciting additional orthogonal field components along the lengths (*L*_*s*1_, *L*_*s*2_) and width (*W*_*s*1_, *W*_*s*2_). By proper tuning of the adjustable parameters, the CP radiating modes are merged resulting in the wide impedance and AR bandwidth.

For the purpose of optimization, the antenna geometry is described using the following parameter vector ***x*** = [*W*_*s*_*W*_*cr*_* W*_*s1r*_*W*_*s*2*r*_* L*_*s*1*r*_* L*_*s*2*r*_* L*_*g*1*r*_* L*_*g*2*r*_* L*_*m*_* d*_*L*_* L*_1*r*_* L*_2_]^*T*^. The parameters with *r*-subscript stand for relative variables (with ranges between zero and one), introduced in order to maintain physical consistency of the structure during the optimization process, e.g., to avoid the ground-plane slot extend beyond the antenna substrate outline, etc. The units for absolute parameters is mm. The relationships between the optimization variables and geometry parameters in Fig. 4 are as follows: *L*_1_ = *L*_1*r*_(*L*_*m*_ + *d*_1_ – *L*_*g*1_) with *d*_1_ = 0.484 mm; *L*_*c*_ = *L*_*cr*_*L*_*s*_; *L*_*g*2_ = *L*_*g*2*r*_*L*_*s*_; *L*_*s*_ = *L*_*m*_ + *d*_1_ + *W*_2_ + *d*_*L*_ with *W*_2_ = 2.16 mm; *L*_*s*1_ = *W*_*s*1r_ (*W*_*s*_/2-*W*_*c*_/2); *L*_*s*2_ = *W*_*s*2r_ (*W*_*s*_/2-*W*_*c*_/2); *W*_*c*_ = *W*_*cr*_*W*_*s*_; *Wg*_1_ = *W*_*s*_/2-*g*-*W*_*m*_/2 with *g* = 0.5 mm, and *W*_*m*_ = 1.35 mm; *W*_*g*2_ = *W*_*s*_/2-*g*-*W*_*m*_/2; *W*_*s*1_ = *L*_*s*1*r*_*L*_*c*_; *W*_*s*2_ = *L*_*s*2*r*_*L*_*c*_.

The antenna of Fig. 4 has been optimized for minimum size using the objective function (3)–(5) and to satisfy the conditions (1) and (2), i.e., |*S*_11_|≤ –10 dB and *AR* ≤ 3 dB within the assumed operational bandwidth. The initial design has been generated as described.

**Figure 5:**
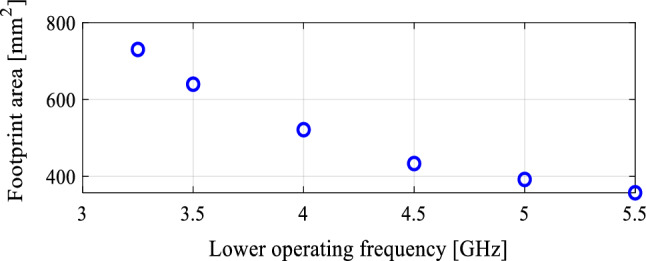
Trade-off designs for the CP antenna of Fig. 4: minimum attainable footprint area versus lower end of the operating bandwidth (minimum value of the upper end equals 7.5 GHz for all cases).

**Figure 6:**
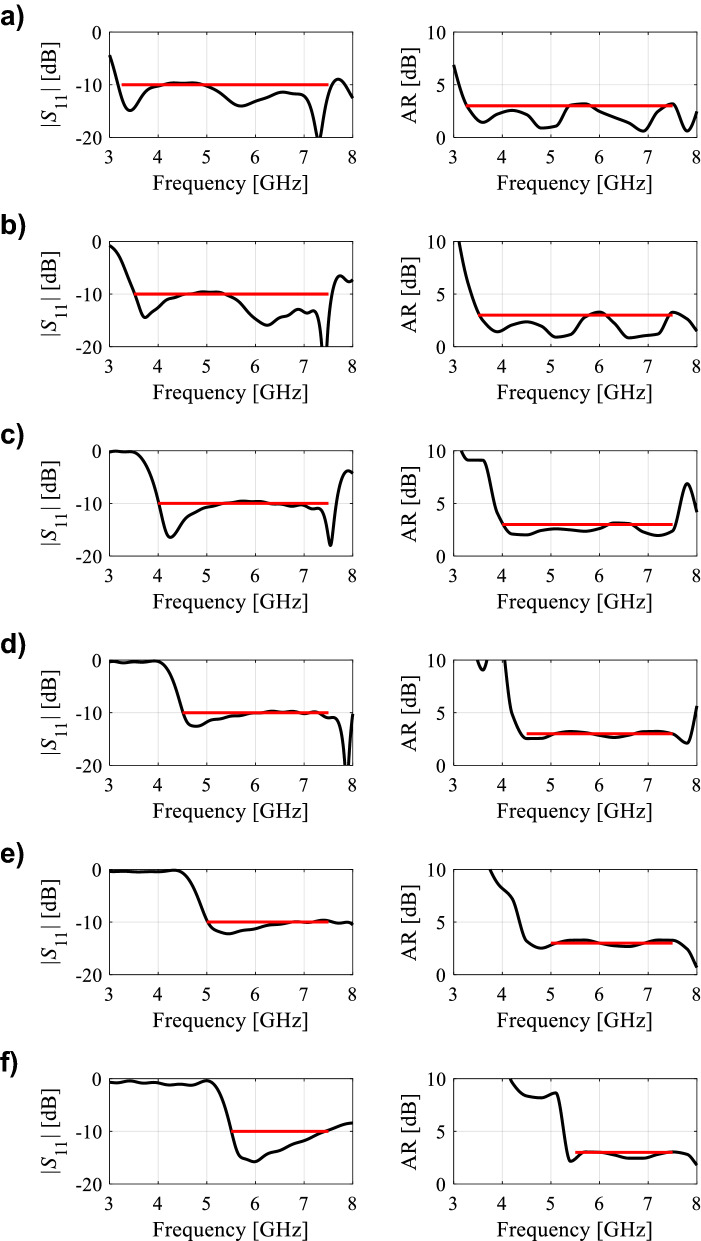
Trade-off designs for the CP antenna of Fig. 4: reflection (left) and axial ratio (right) responses. Horizontal lines represent the target operating bandwidth: (**a**) *f*_*L*_ = 3.25 GHz, (**b**) *f*_*L*_ = 3.5 GHz, (**c**) *f*_*L*_ = 4.0 GHz, (**d**) *f*_*L*_ = 4.5 GHz, (**e**) *f*_*L*_ = 5.0 GHz, (**f**) *f*_*L*_ = 5.5 GHz.

**Figure 7:**
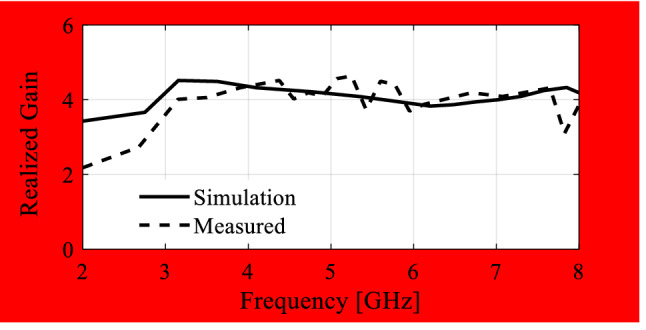
Simulated and measured realized gain of the final design (Example 1).

In the course of the optimization process, there were six designs generated, corresponding to the lower ends of the operating bandwidth *f*_*L*.*k*_ = 3.25 GHz, 3.5 GHz, 4.0 GHz, 4.5 GHz, 5.0 GHz, and 5.5 GHz. In all cases, the upper end of the bandwidth was the same and equal to *f*_*H*_ = 7.5 GHz. Figure 5 shows the obtained Pareto set, illustrating the trade-offs between the antenna size and its operating bandwidth. The geometry parameter values as well as the reflection and axial ratio responses can be found in Table 1 and Fig. 6, respectively. The results indicate that—once the antenna is properly optimized—even a slight narrowing of the operating bandwidth has a profound effect on the antenna size. For example, increasing *f*_*L*_ from 3.25 to 3.5 GHz enables size reduction by over 12%, whereas for *f*_*L*_ = 4.0 GHz, the reduction is as high as almost 30%.The simulated and measured realized gain of the antenna in example 1 is illustrated in Fig. 7. A variation of ± 0.5 dB is observed between the simulated and the measured values, which is a typical error level for the available measurement setup. The average realized gain within the CP operating band is approximately 3.4 dB.

**Table 1:** Trade-off designs for the CP antenna of Fig. 4: geometry parameter values.

Design #	Performance parameters^$^		Geometry parameter values*											
	*f*_*L*_ [GHz]	*A* [mm^2^]	*W*_*s*_	*W*_*cr*_	*W*_*s1r*_	*W*_*s*2*r*_	*L*_*s*1*r*_	*L*_*s*2*r*_	*L*_*g*1*r*_	*L*_*g*2*r*_	*L*_*m*_	*d*_*L*_	*L*_1*r*_	*L*_2_
1	3.25	730	34.4	0.38	0.78	0.64	0.60	0.38	0.41	0.26	15.73	2.87	0.92	7.22
2	3.5	640	32.9	0.34	0.76	0.60	0.48	0.40	0.40	0.23	13.95	2.83	0.85	7.01
3	4.0	521	30.9	0.30	0.79	0.58	0.30	0.42	0.43	0.16	12.16	2.07	0.79	6.74
4	4.5	433	28.7	0.26	0.76	0.57	0.30	0.40	0.43	0.10	10.61	1.82	0.69	5.48
5	5.0	392	27.1	0.24	0.82	0.57	0.28	0.39	0.44	0.12	10.02	1.82	0.61	4.53
6	5.5	357	24.8	0.25	0.90	0.66	0.30	0.39	0.43	0.10	10.02	1.72	0.50	3.57

^$^*f*_*L*_ and *A* stand for the lower end of the operating bandwidth, and the footprint area, respectively.

*Absolute parameters in mm; relative parameters (ending with subscript *r*) unit-less.

Furthermore, rigorous variable adjustment and exploitation of all possible degrees of freedom allow for precise control of both *S*_11_ and *AR* so that at the optimized designs, either reflection or axial ratio characteristics (or both in the majority of cases) conform to the target lower operating frequency. It should be recalled that the optimization process was not concerned about antenna performance beyond *f*_*H*_ so whether antenna characteristics satisfy conditions (1) and (2) therein is just a matter of coincidence.

## Example 2: Wide-slot CP antenna^48^

**Figure 8:**
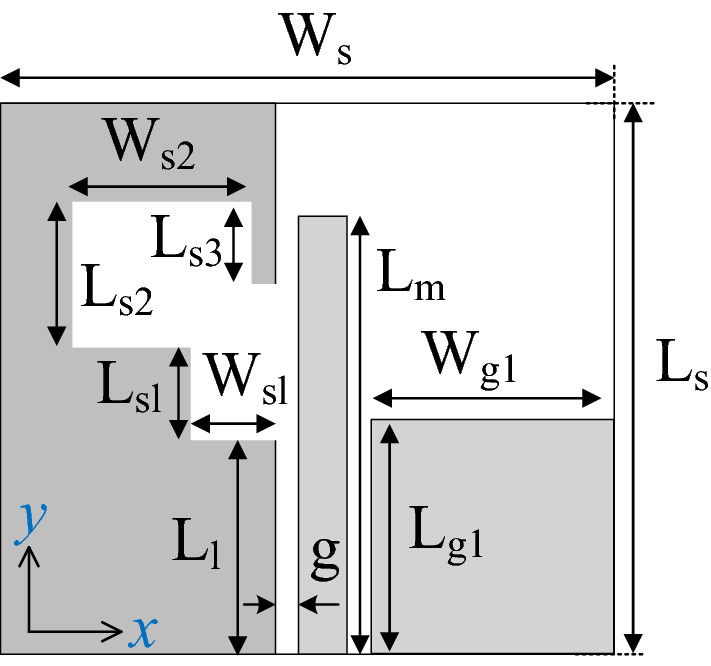
Wide-slot CP antenna^48^: parameterized view.

The second case is the wide-slot antenna^48^ designed on a single-sided Rogers 4003C substrate (*ε*_*r*_ = 3.38, tan*δ* = 0.0027, *h* = 0.813 mm). The antenna geometry is shown in Fig. 8. A combination of microstrip line feeding with geometrically modified coplanar ground planes are used to attain a wide axial ratio and impedance bandwidth. The symmetry of the coplanar ground plan is broken, and a quasi-rectangular loop is formed in the elongated ground plane. The current flow forming a loop is inherently circularly polarized, therefore, the antenna yields circular polarization. The vertical current on the microstrip line and the horizontal current on the shortened coplanar ground plane also contribute to the wide axial ratio bandwidth.

In order to optimize the antenna while ensuring the structural consistency, the geometry is described using the following parameter vector ***x*** = [*L*_1*r*_* L*_*c*1_ *L*_*c*2_ *W*_*c*1*r*_* W*_*c*2*r*_* L*_*mr*_* L*_*g*2*r*_* W*_*g*1_ *d*_*L*_* dx*]^*T*^, which includes several relative variables. The relations between the relative parameters and the antenna dimensions indicated in Fig. 8 are: *L*_1_ = *L*_1*r*_ (*L*_*c*1_ + *L*_*c*2_); *W*_*c*1_ = *W*_*c*1*r*_*W*_*g*1_;*W*_*c*2_ = *W*_*c*2*r*_*W*_*g*1_; *L*_*m*_ = *L*_*mr*_*L*_*s*_; *L*_*s*_ = *d*_*x*_ + *L*_*c*1_ + *L*_*c*2_ + *d*_*L*_; *L*_*g*1_ = *L*_*s*_; *L*_*g*2_ = *L*_*g*2*r*_*L*_*s*_; *W*_*g*2_ = *W*_*g*1_; *W*_*s*_ = *W*_*g*1_ + *W*_*g*2_ + 2* g* + *W*_*m*_ with *g* = 0.6325 and *W*_*m*_ = 1.35 mm. Here, *g* is the coplanar gap and *W*_*m*_ is the width of the microstrip feedline for 50-Ω impedance.

**Figure 9:**
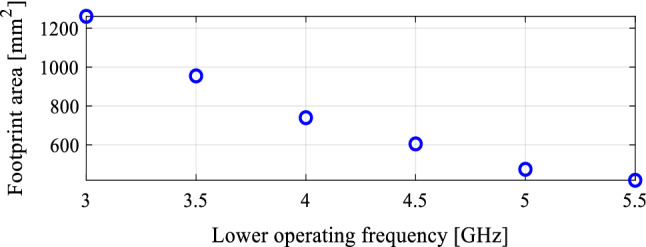
Trade-off designs for the CP antenna of Fig. 7. minimum attainable footprint area versus the lower end of the operating bandwidth (minimum value of the upper end equals 7.5 GHz for all cases).

The antenna of Fig. 7 has been optimized for minimum size using the objective function (3)–(5), and to satisfy the conditions |*S*_11_|≤ –10 dB and *AR* ≤ 3 dB within the assumed operational bandwidth. Six designs were generated, corresponding to the lower ends of the operating bandwidth *f*_*L*.*k*_ = 3.0 GHz, 3.5 GHz, 4.0 GHz, 4.5 GHz, 5.0 GHz, and 5.5 GHz. In all cases, the upper end of the bandwidth was the same and equal to *f*_*H*_ = 7.5 GHz. The obtained Pareto set has been shown in Fig. 9.

**Figure 10:**
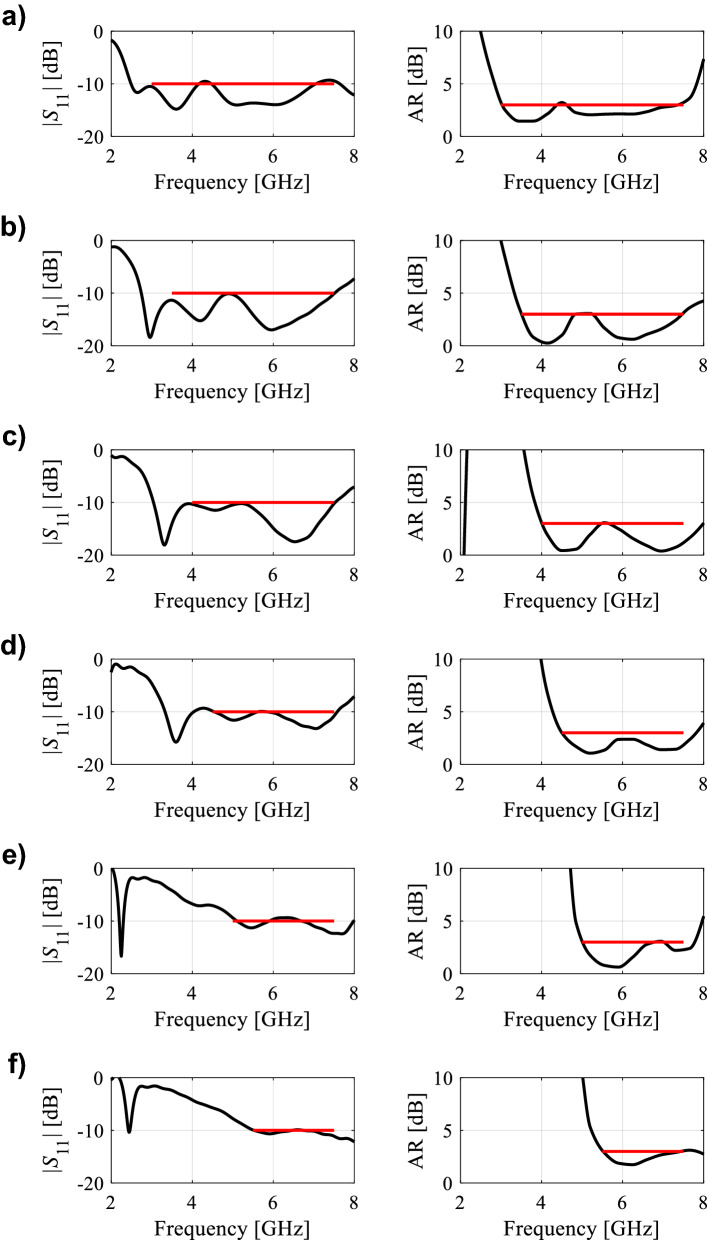
Trade-off designs for the CP antenna of Fig. 7: reflection (left) and axial ratio (right) responses. Horizontal lines represent the target operating bandwidth: (**a**) *f*_*L*_ = 3.0 GHz, (**b**) *f*_*L*_ = 3.5 GHz, (**c**) *f*_*L*_ = 4.0 GHz, (**d**) *f*_*L*_ = 4.5 GHz, (**e**) *f*_*L*_ = 5.0 GHz, (**f**) *f*_*L*_ = 5.5 GHz.

**Figure 11:**
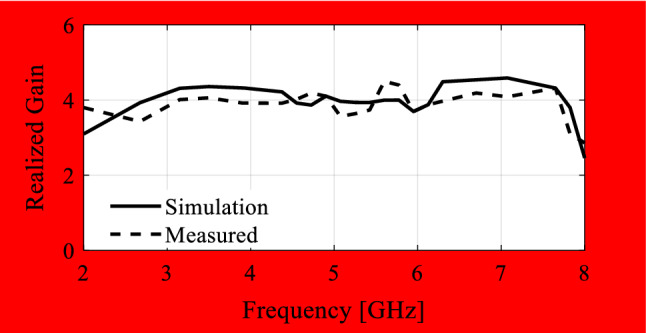
Simulated and measured realized gain of the final design (Example 2).

The geometry parameter values and the reflection and axial ratio responses can be found in Table 2 and Fig. 10, respectively. The results are consistent with those discussed in before. Rigorous optimization of the antenna parameters enables a significant reduction of its physical dimensions, e.g., increasing *f*_*L*_ from 3.0 GHz to 3.5 GHz leads to over 24% reduction of the footprint area, whereas for *f*_*L*_ = 4.0 GHz, the reduction is as high as 41%. The simulated and measured realized gain of the antenna in example 2 is shown in Fig. 11. The measured results shows a variation of ± 0.5 dB between the simulated and the measured data. The average realized gain within the CP operating band is approximately 3.6 dB, and the antenna 3 dB gain bandwidth in the entire operating bandwidth.

**Table 2:** Trade-off designs for the CP antenna of Fig. 8: geometry parameter values.

Design #	Performance parameters^$^		Geometry parameter values*									
	*f*_*L*_ [GHz]	*A* [mm^2^]	*L*_1*r*_	*L*_*c*1_	*L*_*c*2_	*W*_*c*1*r*_	*W*_*c*2*r*_	*L*_*mr*_	*L*_*g*2*r*_	*W*_*g*1_	*dL*	*dx*
1	3.0	1261	0.47	3.01	12.67	0.35	0.90	0.89	0.49	20.85	3.29	9.48
2	3.5	955	0.53	3.07	10.03	0.36	0.89	0.92	0.47	16.92	3.83	9.27
3	4.0	740	0.40	3.05	9.34	0.55	0.90	0.95	0.50	13.37	2.73	10.10
4	4.5	605	0.26	3.01	9.70	0.64	0.90	0.99	0.50	11.81	1.17	9.17
5	5.0	474	0.16	4.06	9.30	0.90	0.90	0.87	0.47	8.86	1.16	8.79
6	5.5	418	0.13	4.04	7.97	0.90	0.90	0.85	0.47	8.44	1.41	8.02

^$^*f*_*L*_ and *A* stand for the lower end of the operating bandwidth, and the footprint area, respectively.

*Absolute parameters in mm; relative parameters (ending with subscript *r*) unit-less.

For this antenna, the impedance bandwidth is typically slightly wider than the AR bandwidth for the first few designs; however, in all cases, either the reflection or axial ratio characteristics conform to the target lower operating frequency. In contrast to the many antenna designs presented in the literature, this antenna maintains a relatively stable bandwidth overlap between the impedance bandwidth and the axial ratio bandwidth.

By closely observing the response of both the performance figures considered in Fig. 9, the impedance matching is well below the − 10 dB reference criteria in the majority of the operating band. Likewise, for the axial ratio, the AR ≤ 3 dB benchmark has been satisfied for all the trade-off designs.

## Example 3: Wide-slot CP antenna^49^

To further demonstrate the effectiveness of the proposed method, a third example is demonstrated^49^. The antenna is implemented on a relatively thin Arlon AD250C substrate (*ε*_*r*_ = 2.5, tan*δ* = 0.0014, *h* = 0.762 mm), and laminated on both sides. A bottom-grounded asymmetrical coplanar waveguide feeding is used for excitation of the antenna with a 50-Ω microstrip line. On the front side, a vertically placed straight microstrip line is coupled with a parasitically placed bracket-shape strip in the horizontal plane. A wideslot is etched in the backside ground plane, and a horizontal strip is protruded from one edge towards the center of the slot. This geometrical configuration of the feedline, the wide slot, and the asymmetrical coplanar ground planes leads to the excitation of orthogonal field components, which results in wide impedance and axial ratio bandwidth.

**Figure 12:**
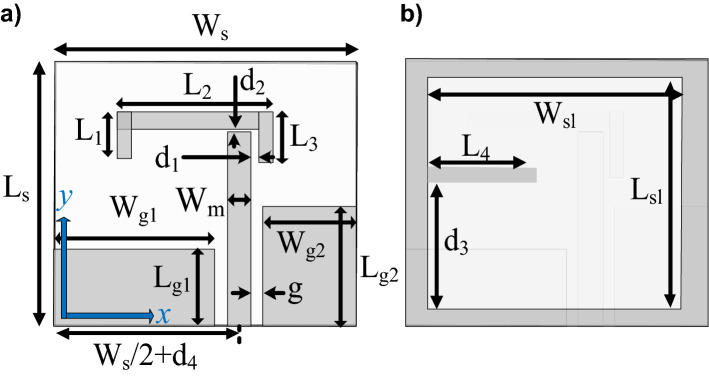
Wide-slot CP antenna^49^, parameterized view: (**a**) front, (**b**) back.

**Figure 13:**
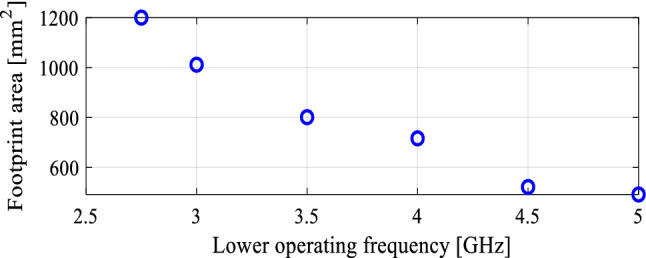
Trade-off designs for the CP antenna of Fig. 10. minimum attainable footprint area versus the lower end of the operating bandwidth (minimum value of the upper end equals 7.5 GHz for all cases).

**Figure 14:**
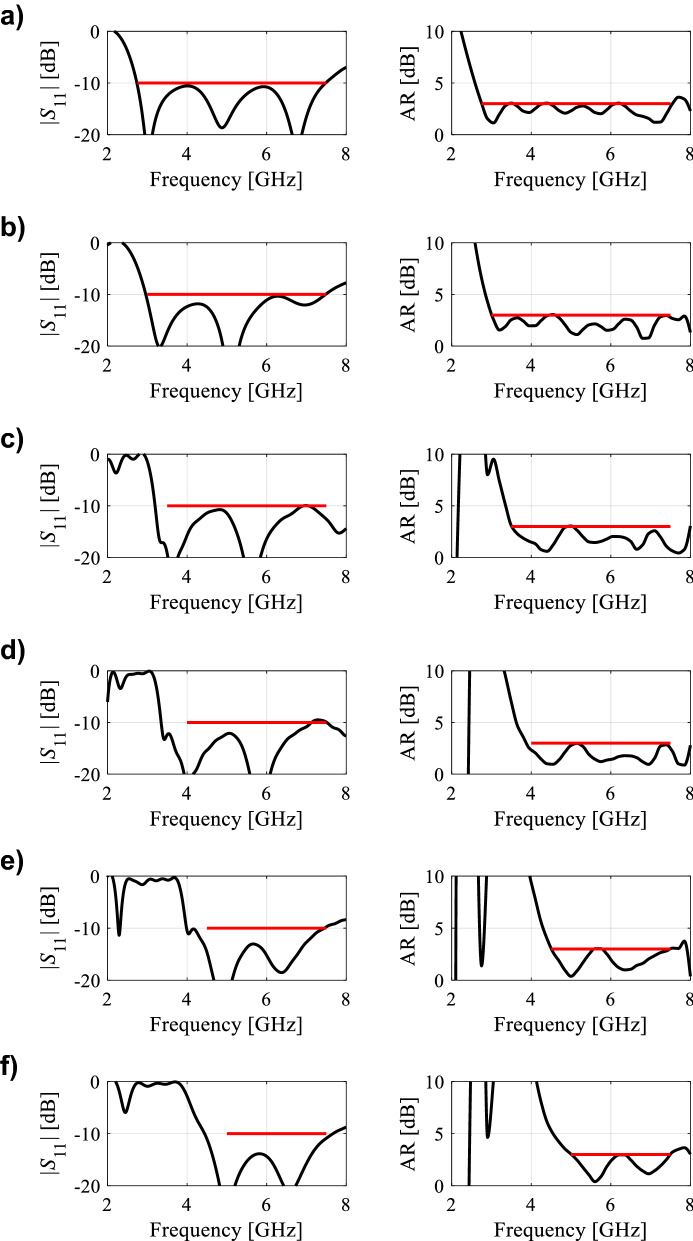
Trade-off designs for the CP antenna of Fig. 10: reflection (left) and axial ratio (right) responses. Horizontal lines represent the target operating bandwidth: (**a**) *f*_*L*_ = 2.75 GHz, (**b**) *f*_*L*_ = 3.0 GHz, (**c**) *f*_*L*_ = 3.5 GHz, (**d**) *f*_*L*_ = 4.0 GHz, (**e**) *f*_*L*_ = 4.5 GHz, (**f**) *f*_*L*_ = 5.0 GHz.

The antenna of Fig. 12 has been optimized using the same setup as for the other examples, i.e., for minimum size while meeting the conditions |*S*_11_|≤ − 10 dB and *AR* ≤ 3 dB within the assumed operational bandwidth. In this case, the following lower ends of the operating bandwidth were used: *f*_*L*.*k*_ = 2.75 GHz, 3.0 GHz, 3.5 GHz, 4.0 GHz, 4.5 GHz, and 5.0 GHz. In all cases, the upper end of the bandwidth was the same and equal to *f*_*H*_ = 7.5 GHz. The obtained trade-off designs have been shown in Fig. 13. The geometry parameter values and the reflection and axial ratio responses can be found in Table 3 and Fig. 14, respectively.

**Table 3:** Trade-off designs for the CP antenna of Fig. 12: geometry parameter values.

Design #	Performance parameters^$^		Geometry parameter values*														
	*f*_*L*_ [GHz]	*A* [mm^2^]	*L*_*s*_	*x*	*x*_*Lm*_	*W*_*g*1_	*x*_*Lg*1_	*x*_*Lg*2_	*x*_1*r*_	*L*_1_	*L*_2_	*L*_3_	*x*_*L*s1_	*x*_*W*s1_	*x*_*L*4_	*dxr*	*x*_3_
1	2.75	730	29.98	2.74	0.72	18.62	0.10	0.35	0.88	2.41	15.56	2.65	0.95	0.71	0.31	0.02	− 0.78
2	3.0	640	27.79	2.01	0.73	16.80	0.16	0.38	0.86	2.45	15.50	2.68	0.95	0.73	0.30	0.03	− 0.74
3	3.5	521	25.37	2.00	0.74	14.40	0.11	0.37	0.91	2.54	14.30	2.25	0.95	0.75	0.31	0.02	− 0.87
4	4.0	433	24.28	2.03	0.73	13.36	0.12	0.38	0.91	2.52	13.85	2.26	0.95	0.76	0.30	0.02	− 0.83
5	4.5	392	20.99	2.02	0.74	11.04	0.14	0.42	0.93	2.58	11.71	2.09	0.95	0.83	0.30	0.05	− 0.73
6	5.0	357	20.00	2.11	0.74	10.90	0.14	0.43	0.91	2.57	12.20	2.26	0.93	0.81	0.31	0.04	− 0.65

^$^*f*_*L*_ and *A* stand for the lower end of the operating bandwidth, and the footprint area, respectively.

*Absolute parameters in mm; relative parameters (ending with subscript *r*) unit-less.

**Figure 15:**
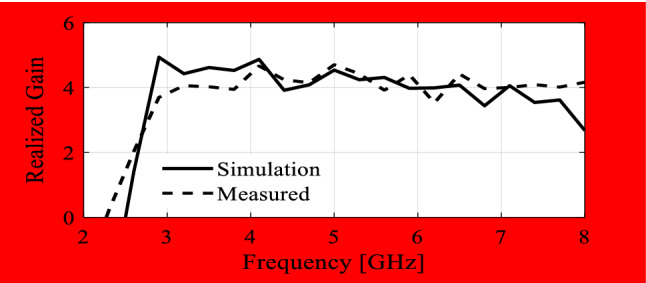
Simulated and measured realized gain of the final design (Example 3).

Again, the results are consistent with those obtained in the previous sections. As before, proper optimization allows for a considerable size reduction of the antenna structure. Here, reducing the bandwidth from below by just 0.25 GHz results in an almost 20% reduction of the footprint, which doubles when *f*_*L*_ = 3.5 GHz. At the same time, both the impedance bandwidth and axial ratio bandwidth conform to the assumed specifications. All the trade-off designs persuasively satisfy the |*S*_11_|≤ –10 dB and AR ≤ 3 dB criteria for the impedance matching and axial ratio.The simulated and measured realized gain of the antenna in example 3 is shown in Fig. 15. Based on the measured results, the average realized gain within the CP operating band is approximately 3.5 ± 0.5 dBiC , and the antenna 3 dB gain bandwidth in the entire operating bandwidth.

## Benchmarking

A comprehensive benchmarking has been performed in the following Table 4. The four vital parameters are compared with a large set of similar designs. The lowest frequency of all the refrence designs is in the sub-6 GHz rang.The results clearly show that the antennas developed through the proposed multi-objective optimization approach yield excellent performance in terms of AR and S_11_and outperform all the widely adopted antenna design techniques. All three antenna retains an average realized gain of 3.3 ± 0.5 dBiC, in the entire operating frequency along with stable bidirectional radiation patterns and miniature size in the sub-wavelength range. The size of the antenna is calculated at the lowest operating frequencies of the reference antennas in terms of the free-space wavelength. All designs considered and optimized in this paper maintain a 3 dB gain-bandwidth in the entire CP operating band of the antennas.

**Table 4:** Comparison with state-of-the-art CP antennas.

References	%AR	%BW	Size [λ_o_^2^]	Peak Realized Gain (dBiC)	Substrate (*h* (mm), *ε*)
^50^	40.8	47.4	0.16	5.3	GLS (1.0, 5.5)
^51^	41.3	84	0.28	3.4	RT (1.6, 2.2)
^52^	27.45	71.63	0.42	2.5	FR4 (1.6,4.4)
^53^	39.4	57	0.78	–	AD (0.762, 2.5)
^54^	40	90.2	0.28	4.5	FR4 (1.6,4.4)
^55^	26.9	27	1.02	4–5	FR4 (1.6,4.4)
^56^	22	37	0.89	5	RT (0.8, 4.4)
^57^	42	55.5	0.15	3.5	FR4 (1,4.4)
^58^	42	42.6	0.17	3.4	RT (0.81, 3.38)
Proposed 1	97%	97%	0.07	3.9	RO (0.813, 3.38)
Proposed 2	97%	97%	0.09	4.1	AD (0.762, 2.5
Proposed 3	98%	98%	0.13	4.3	AD (0.762, 2.5)

## Conclusion

The paper proposed a rigorous algorithmic framework for generating the families of minimum-size designs of broadband circularly-polarized antennas. Our methodology is based on inverse modeling techniques for generating the starting points for further tuning, and expedited refinement procedures involving gradient search with sparse sensitivity updates. It allows for reliable identification of design trade-offs between the antenna physical dimensions (here, footprint area), and the lower end of the operating frequency, which effectively solves a multi-objective optimization task with respect to these two criteria. Meticulous treatment of all relevant antenna parameters allows for finding the designs that simultaneously ensure the satisfaction of the impedance matching condition (|*S*_11_|≤ –10 dB), and the axial ratio requirements (AR ≤ 3 dB) over the prescribed bandwidth while yielding the structure of minimum possible size.

The presented approach has been comprehensively demonstrated using three examples of broadband wide-slot CP antennas. In each case, six trade-off designs have been generated for the operating bandwidths ranging from 2.75 to 3.25 GHz (case dependent) to 7.5 GHz. The obtained results indicate that even a slight reduction of the bandwidth may result in a considerable reduction of the antenna size of up to 30–40% depending on the structure. From the perspective of practical antenna design, the knowledge of attainable miniaturization ratio under the prescribed operating conditions is instrumental for selecting the geometrical solutions, especially for space-limited applications. Furthermore, the availability of size-bandwidth trade-offs allows for meaningful comparison of alternative antenna topologies in the context of specific applications. Finally, it should be emphasized that the presented algorithmic framework is quantitatively different from conventional multi-objective approaches (including surrogate-assisted methods) because it is entirely deterministic, does not involve computationally heavy nature-inspired procedures, allows for handling multiple design constraints, and enables the accomplishment of the Pareto set rendition task within practically acceptable timeframes.Also, the presented algorithm is computationally efficient. The average cost of identifying a trade-off design is only about 70 EM simulations of the antenna at hand, which is due to applying the sequential approach (the previously-found trade-off design being the starting point for the next one), and the acceleration mechanisms.
